# Supplementary material for: HTLV-1 infection of donor-derived T cells might promote acute graft-versus-host disease following liver transplantation
Source: Nat Commun. 2022 Nov 30;13:7368. doi: 10.1038/s41467-022-35111-w (PMC9712688; doi:10.1038/s41467-022-35111-w)
Supplement: Supplementary file 1 — Supplementary Information [file 41467_2022_35111_MOESM1_ESM.pdf]

HTLV-1 infection of donor-derived T cells might promote acute Graft-versus-host disease following liver transplantation

Chuan Shen[1#], Yiyang Li[2#], Boqian Wang[2#], Zhipeng Zong[1#], Tianfei Lu[3], Nokuzola Maboyi[2], Yuxiao Deng[1], Yongbing Qian[1], Jianjun Zhang[1*], Xianting Ding[2*], and Qiang Xia[1*]

[1]Department of Liver Surgery, Renji Hospital, Shanghai Jiao Tong University School of Medicine, Shanghai Jiao Tong University, 160 Pujian Road, Shanghai, 200127, China

[2]State Key Laboratory of Oncogenes and Related Genes, Institute for Personalized Medicine, School of Biomedical Engineering, Shanghai Jiao Tong University, 1954 Huashan Road, Shanghai, 200030, China

[3]Abdominal Organ Transplantation Department, Ruijin Hospital, Shanghai Jiao Tong University School of Medicine, Shanghai Jiao Tong University, 197 Ruijin Road, Shanghai, 20025, China

[#]Equal contribution.

[*]Co-correspondence.

Correspondence:

Jianjun Zhang. Department of Liver Surgery, Renji Hospital, Shanghai Jiao Tong University School of Medicine, 160 Pujian Road, Shanghai, 200127, China.

Email: zhangjianjun@renji.com;

Xianting Ding. State Key Laboratory of Oncogenes and Related Genes, Institute for Personalized Medicine, School of Biomedical Engineering, Shanghai Jiao Tong University, 1954 Huashan Road, Shanghai, 200030, China

Email: dingxianting@sjtu.edu.cn;

Qiang Xia. Department of Liver Surgery, Renji Hospital, Shanghai Jiao Tong University School of Medicine, 160 Pujian Road, Shanghai, 200127, China.

Email: xiaqiang@medmail.com.cn.

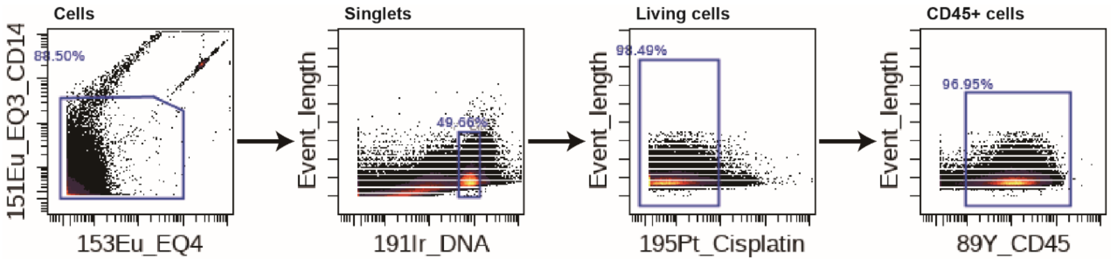

Figure S1. Manual gating scheme.

A typical gating scheme was used to define classical immune cell types. A representative sample was used to display the gating scheme.

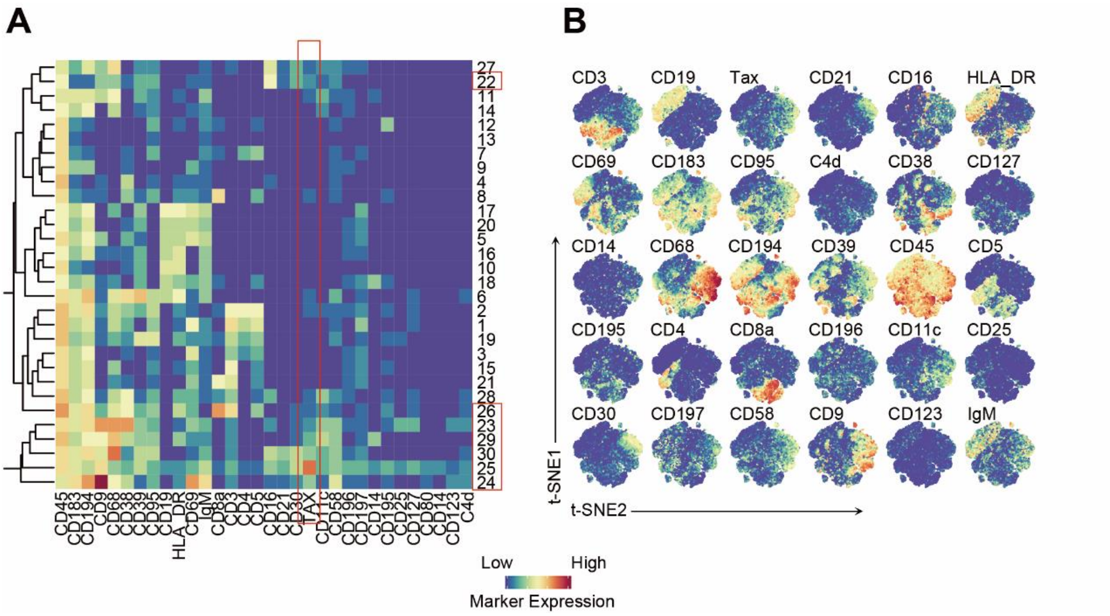

Figure S2. The comprehensive cell phenotypes of 30 clusters.

A. A heatmap depicts the median of respective ArcSinh-transformed cell marker expression values of 30 phenotypic subpopulations. No. 22, No. 23, No. 24, No. 25, No. 26, No. 29, and No. 30 clusters showed positive Tax expression marked in red sold box. The color bar signified the ArcSinh-transformed expression intensity of proteins. **B.** The colors of the cells in collective t-SNE represent ArcSinh-transformed expression values for a given marker. The color scale signified the expression intensity of Collective t-SNE was used to identify 30 phenotypic subpopulations.

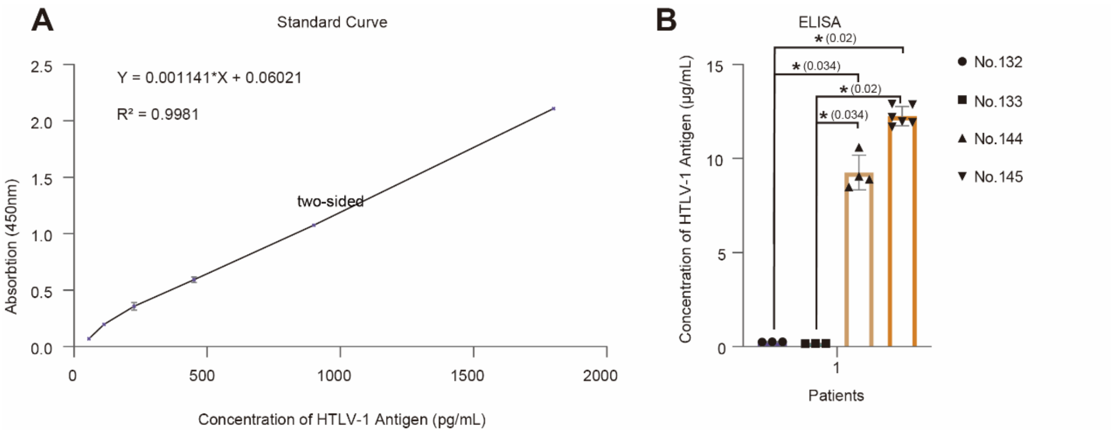

Figure S3. HTLV-1 antigen concentrations detected by ELISA.

A. Standard curves for HTLV-1 antigen concentration determined by ELISA. **B.** HTLV-1 antigen concentrations in serums of two negative controls (N=3) and two aGVHD patients (N=3). Error bars indicate mean ± s.d. *$p < 0.05$. Statistical analysis was performed using two-sided unpaired Mann-Whitney test.

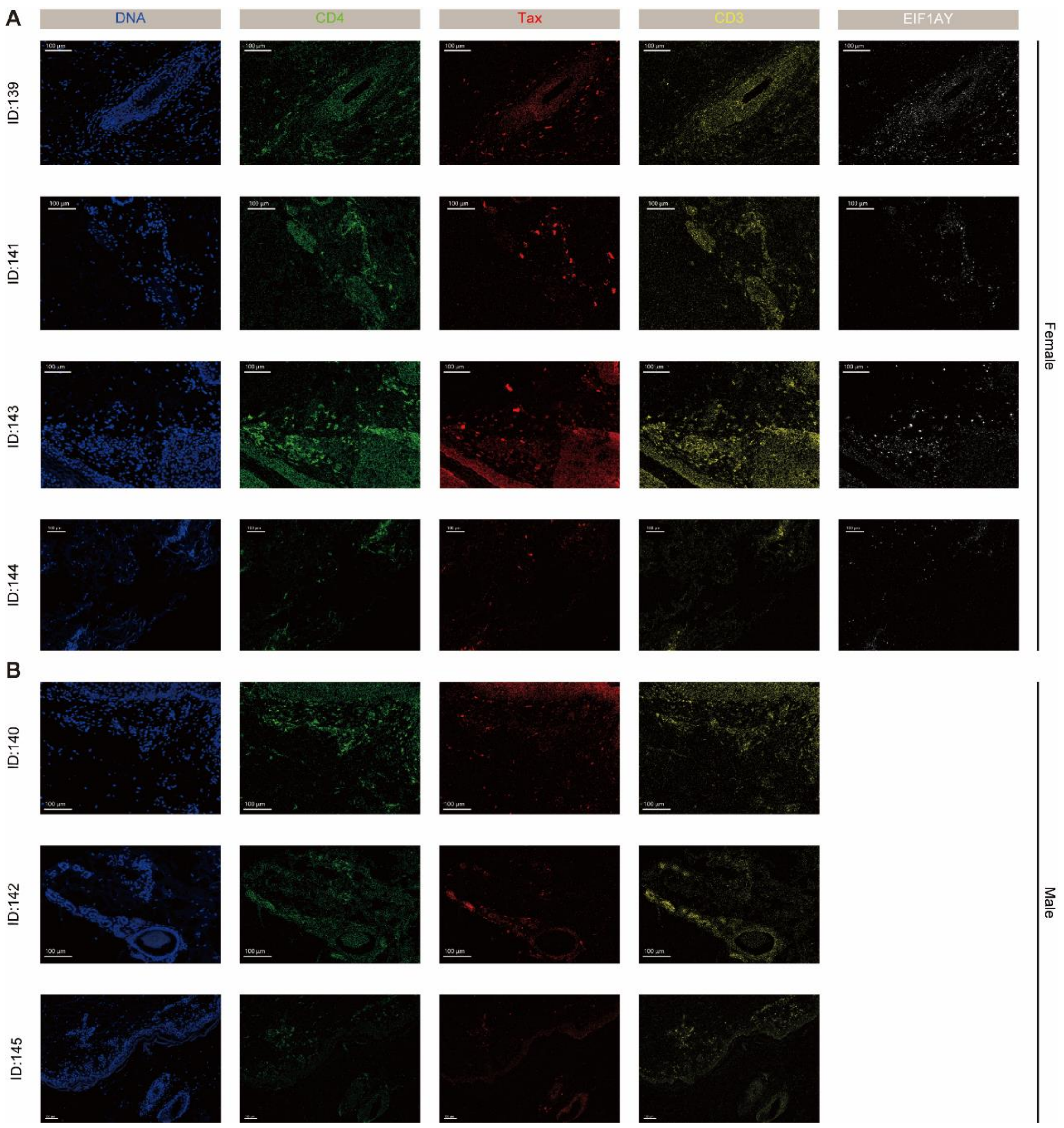

Figure S4. Single color images of "Triple positive T cells". Representative IMC images of two independently stained skin tissues from (A) 4 female aGVHD patients (ID 139, 141, 143 and 144) and (B) 3 male aGVHD patients (ID 140, 142, and 145). Displayed channels for each column were: DNA (blue), CD4 (green), Tax (red), CD3 (yellow), and EIF1AY (white). Scale bar =100 µm.

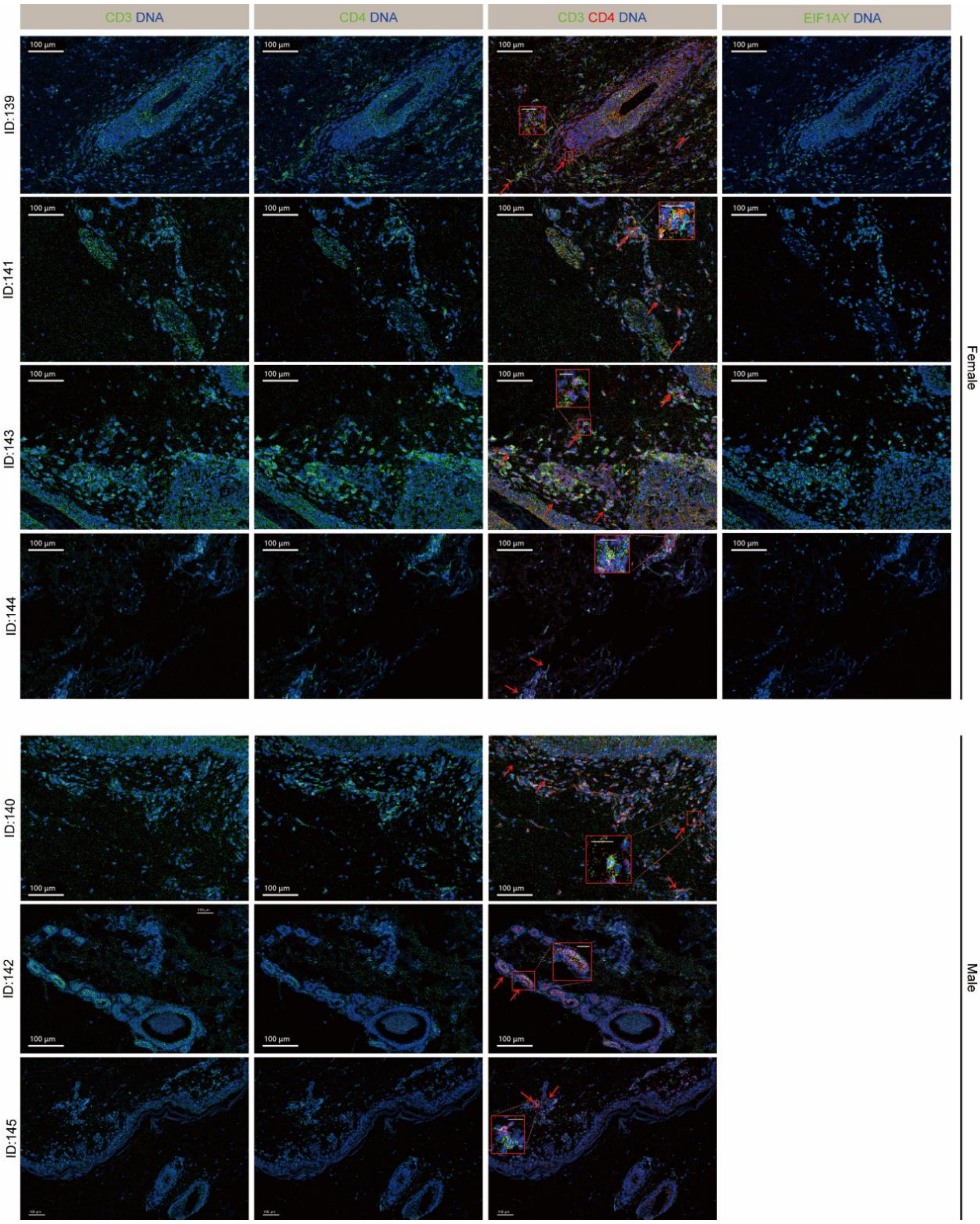

Figure S5. $CD4^+$ T cells detected on aGVHD skin tissues by IMC. Representative IMC images of two independently stained skin tissues from seven aGVHD patients showed the overlap of DNA (blue) and CD3, CD4, and EIF1AY. Displayed channels for each column were: CD3 (green)/ DNA (blue); CD4 (green)/ DNA (blue); CD3 (green)/CD4 (red)/DNA (blue); EIF1AY (green)/DNA (blue). Red arrows pinpointed the $CD3^+CD4^+$T cells position, and red box showed the magnified view. Scale bar =100 μm for landscape view and scale bar =20 μm for magnified view.

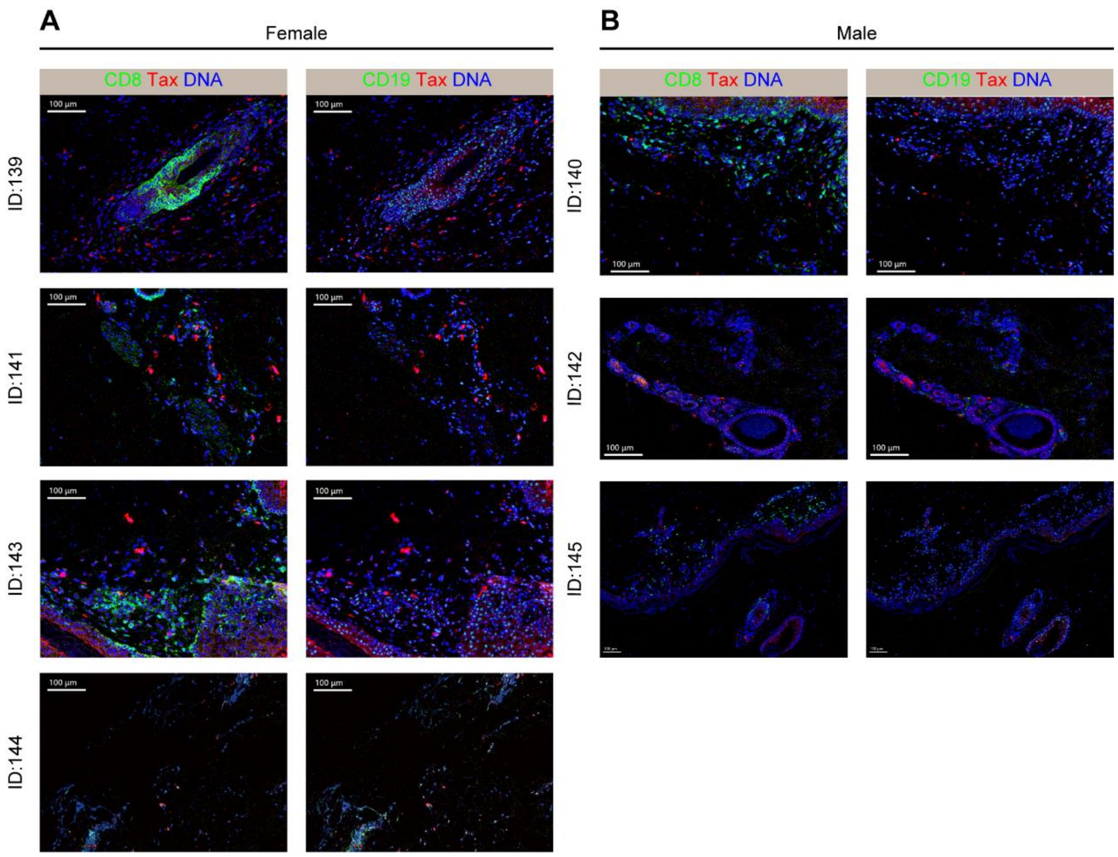

Figure S6. Immune cells other than CD4+ T cells were unrelated with Tax+ in aGVHD patients. Representative IMC images of two independently stained skin tissues from (A) four female and (B) three male aGVHD patients showed the overlap of Tax (red), DNA (blue) and different immunophenotypic markers. The same markers were shown in images in each column. Displayed channels for each column in (A) and (B) were: CD8 (green)/ Tax (red)/DNA (blue) and CD19 (green)/ Tax (red)/DNA (blue). Scale bar =100 µm.

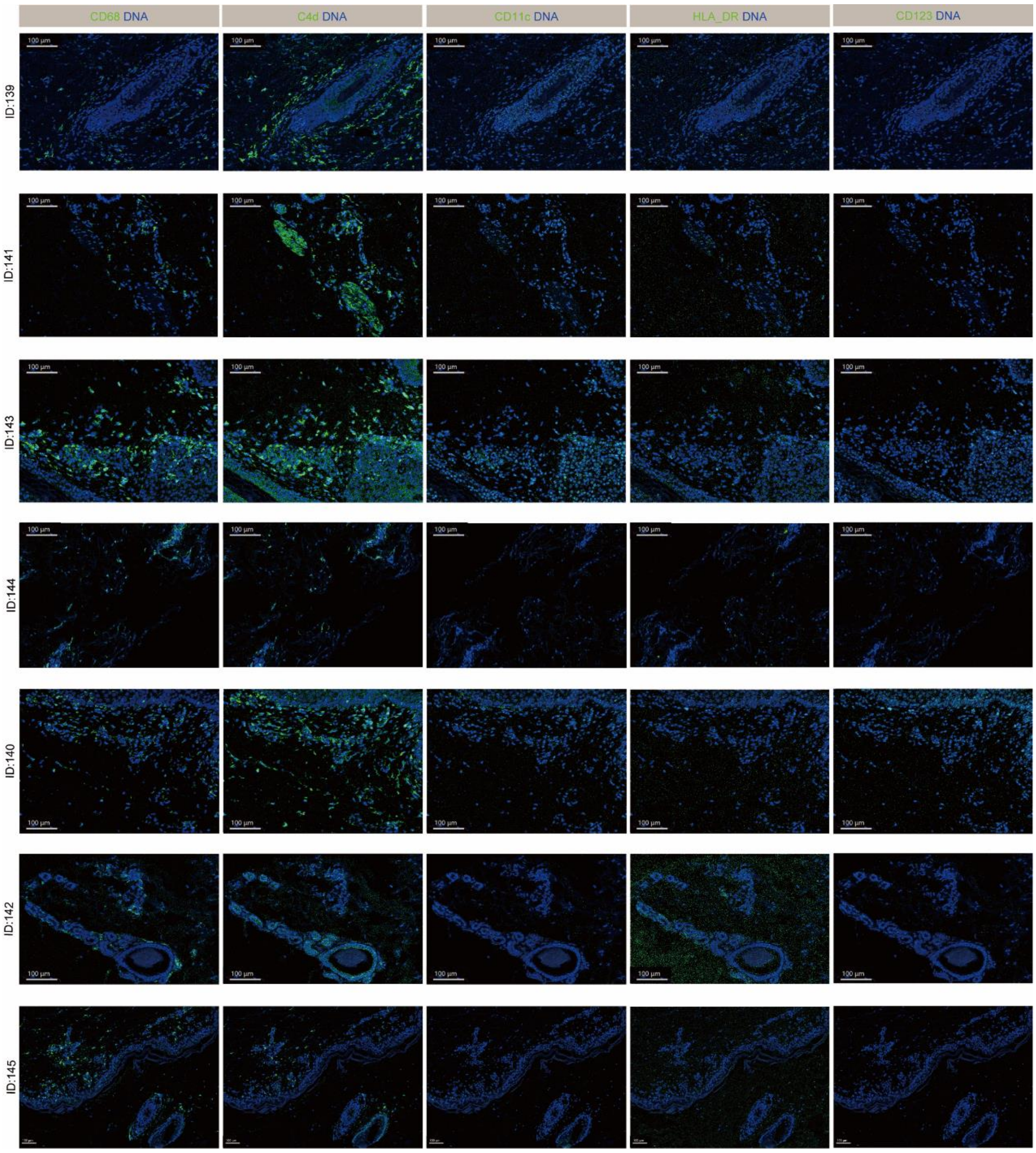

Figure S7. Single marker signals on aGVHD skin tissues by IMC. Representative IMC images of two independently stained skin tissues from seven aGVHD patients showed the overlap of DNA (blue) and five different markers (green), inlcuding CD68, C4d, CD11c, HLA_DR, and CD123. Scale bar =100 μm.

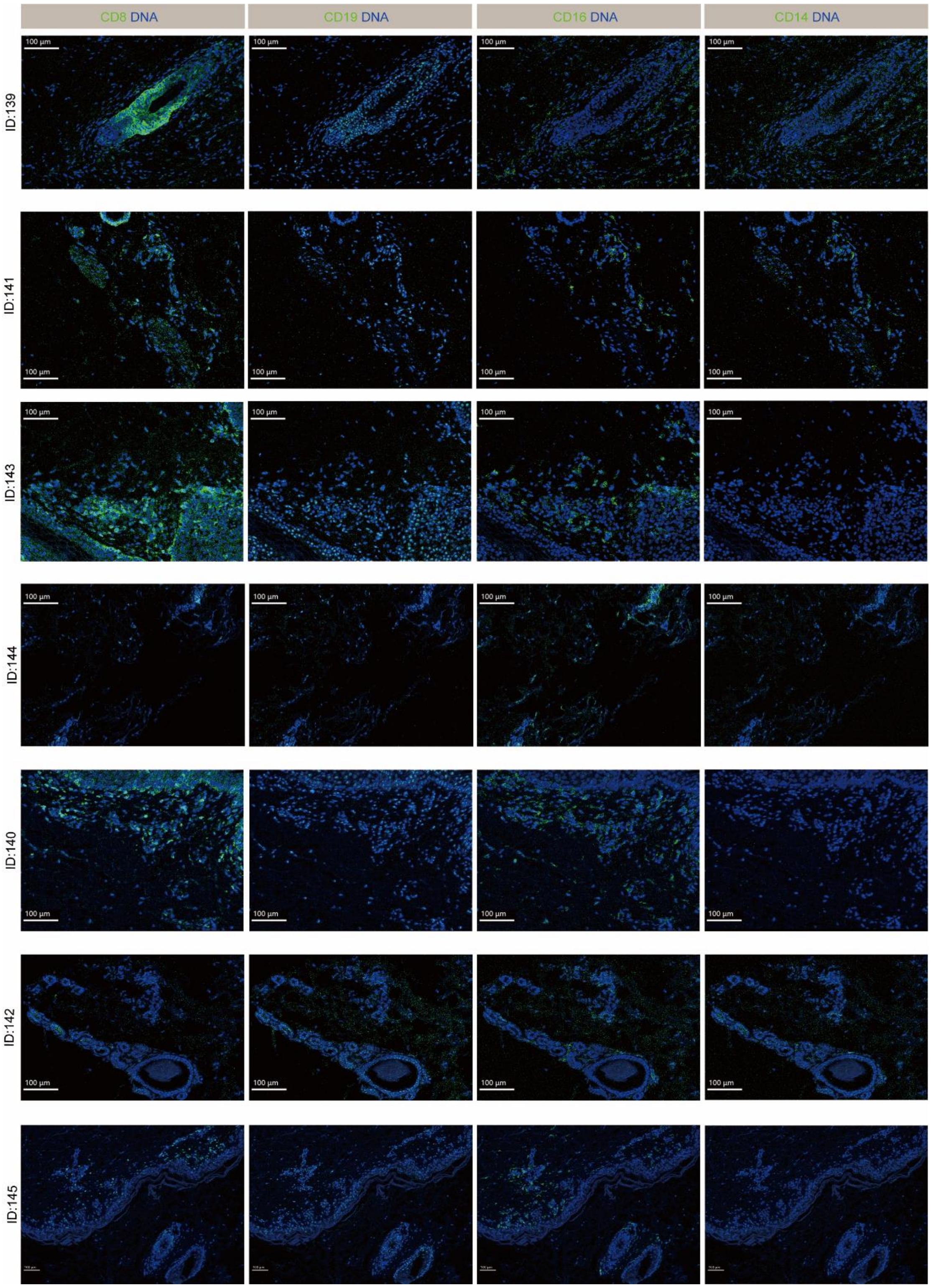

Figure S8. Single marker signals on aGVHD skin tissues by IMC. Representative IMC images of two independently stained skin tissues from seven aGVHD patients showed the overlap of DNA (blue) and four different markers (green), inlcuding CD8, CD19, CD16, and CD14. Scale bar =100 μm.

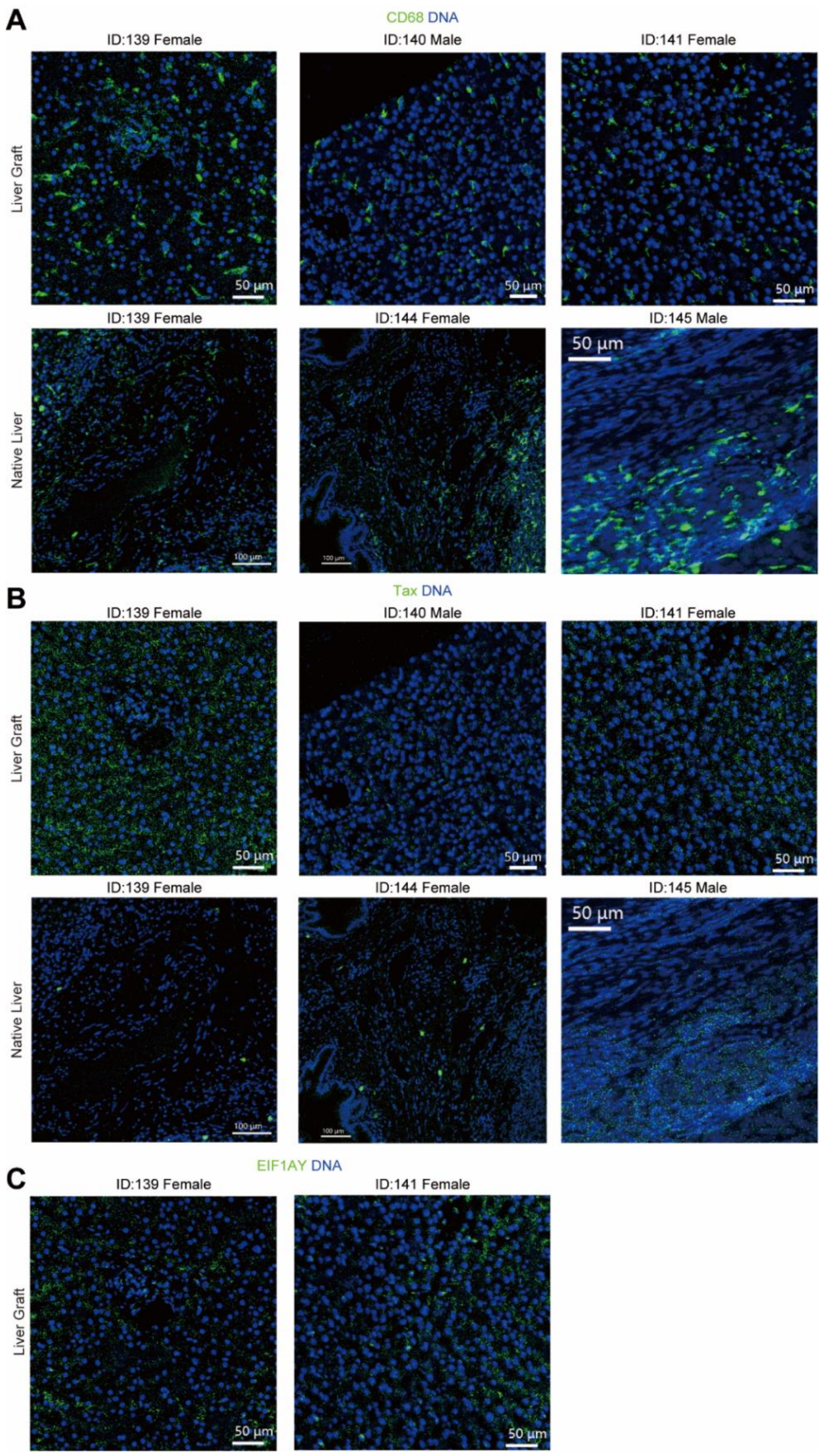

Figure S9. Single marker signals on liver graft, native liver, and bone marrow samples from aGVHD patients by IMC. Representative images of two independently stained liver graft and native liver showed overlap of (A) DNA (blue)/CD68 (green); (B) DNA (blue)/Tax (green); (C) EIF1AY (green)/DNA (blue). Each row of figures displayed the same tissue type.

Table S1 Patients' information profile.

ID	Group	Sex	Age	Diagnosis	Sample type	HTLV-1 detection method				HTLV-1 detection summary
						IMC	Targetseq	CyTOF	ELISA	
145	GVHD	Male	41	aGVHD After OLT	FFPE of skin, native liver; Blood	+	NA	NA	+	+
144	GVHD	Female	62	aGVHD After OLT	FFPE of skin, native liver; Blood	+	NA	NA	+	+
143	GVHD	Female	47	aGVHD After OLT	FFPE of skin	+	NA	NA	NA	+
142	GVHD	Male	50	aGVHD After OLT	FFPE of skin	+	NA	NA	NA	+
141	GVHD	Female	54	aGVHD After OLT	Tissue/FFPE of skin, native/donor liver; Blood	+	+	+	NA	+
140	GVHD	Male	45	aGVHD After OLT	Tissue/FFPE of skin, native/donor liver; Blood	+	NA	+	NA	+
139	GVHD	Female	63	aGVHD After OLT	Tissue/FFPE of skin, native/donor liver; Blood	+	+	+	NA	+
138	Control	Male	48	Normal recovery After OLT	Tissue of donor liver; Blood	NA	-	-	NA	-
137	Control	Male	59	ACR After OLT	Tissue of donor liver; Blood	NA	-	-	NA	-
136	Control	Male	23	HCC recurrence After OLT	Tissue of lung; Blood	NA	-	-	NA	-
135	Control	Female	31	IFI After OLT	Tissue of heart; Blood	NA	-	-	NA	-
134	Control	Male	60	AIH Recipient Before OLT	Tissue of native liver; Blood	NA	-	NA	NA	-
133	Control	Female	32	ACR After OLT	Tissue of donor liver; Blood	NA	-	-	NA	-
132	Control	Male	42	ACR After OLT	Tissue of donor liver; Blood	NA	-	-	NA	-
131	Control	Male	37	Normal recovery After OLT	Tissue of donor liver; Blood	NA	-	-	NA	-
130	Control	Male	52	HCC Recipient Before OLT	Tissue of native liver; Blood	NA	-	NA	NA	-
129	Control	Female	46	ACR After OLT	Tissue of donor liver; Blood	NA	-	-	NA	-
128	Control	Male	56	New hepatitis B After OLT	Tissue of donor liver; Blood	NA	-	-	NA	-
127	Control	Male	67	HCC Recipient Before OLT	Tissue of native liver; Blood	NA	-	NA	NA	-
126	Control	Male	57	HBV Cirrhosis Recipient Before OLT	Tissue of native liver; Blood	NA	-	NA	NA	-
125	Control	Male	36	HCC Recipient Before OLT	Tissue of native liver; Blood	NA	-	NA	NA	-
124	Control	Male	57	Infection After OLT	Tissue of skin; Blood	NA	-	-	NA	-
123	Control	Male	54	HBV Cirrhosis Recipient Before OLT	Tissue of native liver; Blood	NA	-	NA	NA	-
122	Control	Male	22	PTLD After OLT	Tissue of donor liver/ileum; Blood	NA	-	-	NA	-

NA: method not applied

+: HTLV-1 infection was detected
-: HTLV-1 infection was not detected
Abbreviation
HCC: hepatocellular carcinoma
OLT: orthotopic liver transplantation
AR: acute rejection
IFI: invasive fungal infection
AIH: autoimmune hepatitis

Table S2. Clinical information of the 400 patients in HTLV-1 screening cohort.

Characteristics of the sample	
Demographics	
Male/female (n)	273/147
Age (years)	38.3 (0.25-82)
Body mass index (kg/m^2)	21 (11-31)
Concomitant diseases	
Hypertension (n)	37 (9.25%)
Diabetes (n)	73 (18.25%)
Preoperative diagnosis (n)	
Benign end-stage liver disease (n)	78 (19.5%)
Malignant liver disease (n)	175 (43.75%)
Congenital biliary disease (n)	78 (19.5%)
Hereditary metabolic disorder (n)	11 (2.75%)
Complications after liver transplantation (n)	31 (7.75%)
Other diseases (n)	16 (4%)
Liver donors (n)	11 (2.75%)
Sample collection (n)	
One week before operation (n)	149 (37.25%)
Onset time of the complication after operation (n)	251 (62.75%)

Table S3. Virus whole genome sequence acquired from the NCBI GenBank.

VIRUS	HHV-6A	EBV	CMV	ADENOVIRUS	PARVOVIRUS	PARVOVIRUS	HTLV-2	HTLV-1	HTLV-4
**TARGETCHR TARGET**	NC 00159321	AP015177320	NC 00235646	J01917.35937	M2971479	M2971479	NC 008952	NC 008507	NC 018791
**COVERAGE LENGTH (BP)**	157092	176326	3329	35676	383	456	8933	8502	8791
**COVERAGE RATE (%)**	98·6	99·44	99·17	99·27	79·96	95·2	99·79	99·94	100
**NCBI DATABASE LINK**	HHV-6A：https://www.ncbi.nlm.nih.gov/nuccore/1276863980	EBV: https://www.ncbi.nlm.nih.gov/nuccore/AP015016.1	CMV: https://www.ncbi.nlm.nih.gov/nuccore/NC_006273.2	ADV: https://www.ncbi.nlm.nih.gov/nuccore/J01917.1	B19-3’: https://www.ncbi.nlm.nih.gov/nuccore/M29711.1	B19-5’: https://www.ncbi.nlm.nih.gov/nuccore/M29710.1	HTLV-2: https://www.ncbi.nlm.nih.gov/nuccore/9626726	HTLV-1: https://www.ncbi.nlm.nih.gov/nuccore/9626453	HTLV-4: https://www.ncbi.nlm.nih.gov/nuccore/219552908
**BP SS/C-DNA**	159321	177320	235646	35937	479	479	8952	8507	8791

Table S4. Antibody list of CyTOF panel.

PBMC Primary Antibodies						
**Markers**	**Tag**	**Final conc· [μg/mL]**	**Clone**	**Reactivity**	**Vendor**	**Mass**
CD45	89	1	HI30	Human	Fluidigm	Y
CD196 (CCR6)	141	1	G034E3	Human	Fluidigm	Pr
CD19	142	1	HIB19	Human	Fluidigm	Nd
CD5	143	1	UCHT2	Human	Fluidigm	Nd
CD195 (CCR5)	144	1	NP-6G4	Human	Fluidigm	Nd
CD4	145	1	RPA-T4	Human	Fluidigm	Nd
CD8a	146	1	RPA-T8	Human	Fluidigm	Nd
CD11c	147	2	EP1347Y	Human	Abcam	Sm
CD25 (IL-2R)	149	1	2A3	Human	Fluidigm	Sm
CD14	151	1	M5E2	Human	Fluidigm	Eu
CD3	152	2	UCHT1	Human	Biolegend	Sm
CD30	154	2	CON6D/ C2	Human	Invitrogen	Gd
CD68	155	2	KP1	Human/ Mouse	Invitrogen	Gd
CD21	156	2	2G9	Human	Invitrogen	Gd
CD194 (CCR4)	158	1	205410	Human	Fluidigm	Gd
CD197 (CCR7)	159	1	G043H7	Human	Fluidigm	Tb
CD39	160	1	A1	Human	Fluidigm	Gd
CD80 (B7-1)	161	1	2D10.4	Human	Fluidigm	Dy
CD69	162	1	FN50	Human	Fluidigm	Dy
CD183 (CXCR3)	163	1	G025H7	Human	Fluidigm	Dy
CD95	164	1	DX2	Human	Fluidigm	Dy
C4d	166	2	LH61	Human	Invitrogen	Er
CD38	167	1	HIT2	Human	Fluidigm	Er
CD127 (IL-7Ra)	168	1	A019D5	Human	Fluidigm	Er
TAX	170	2	1A3	HTLV-1	Abcam	Er
CD9	171	1	SN4 C3-3A2	Human	Fluidigm	Yb
IgM	172	1	MHM-88	Human	Fluidigm	Yb
CD123	173	2	Monoclonal Mouse IgG1	Human	R&D Systems	Yb
HLA-DR	174	1	YE2/36 HLK	Human	Fluidigm	Yb
CD58 (LFA-3)	176	1	TS2/9	Human	Fluidigm	Yb
CD16	209	1	3G8	Human	Fluidigm	Bi

Table S5. Antibody list of IMC panel.

Tissue antibodies						
**Markers**	**Tag**	**Final conc. [μg/mL]**	**Clone**	**Reactivity**	**Vendor**	**Mass**
CD16	145	10	EPR16784	Human/Rat	Abcam	Nd
CD11c	147	10	EP1347Y	Human	Abcam	Sm
IgG	149	5	IG507R	Human	Abcam	Sm
Collagen	151	10	Polyclonal	Human/Rat/ Mouse	Abcam	Eu
CD3	152	2	Polyclonal	Human/Rat/ Mouse	Abcam	Sm
CD14	153	10	4B4F12	Human	Abcam	Eu
CD30	154	2·5	CON6D/ C2	Human	Invitrogen	Gd
CD68	155	1·25	KP1	Human/ Mouse	Invitrogen	Gd
CD21	156	20	2G9	Human	Invitrogen	Gd
α-SMA	158	0·5	1A4	Human/Rat/ Mouse	Abcam	Gd
HLA-DR	160	5	YE2/36 HLK	Human	Invitrogen	Dy
CD8a	162	5	C8/468+C 8/144B	Human	Abcam	Dy
CD4	164	10	EPR6855	Human	Abcam	Dy
C4d	166	1	LH61	Human	Invitrogen	Er
EIF1AY	169	10	Polyclonal	Human/Rat	Abcam	Tm
TAX	170	10	1A3	HTLV	Abcam	Er
CD38	171	5	EPR4106	Human	Abcam	Yb
CD123	173	10	Monoclon al Mouse I gG1	Human	R&D Systems	Yb
CD19	176	5	6OMP31	Human/Rat/ Mouse	Invitrogen	Yb

Table S6. Characteristic phenotype of aGVHD group specific clusters.

Cluster	No.22	No.23	No.24	No.25	No.26	No.29	No.30
**Phenotype (+)**	CD16<br>CD194<br>CD21<br>CD39<br>CD38	CD11c<br>CD127<br>CD16<br>CD194<br>CD38<br>CD39<br>CD58<br>CD69	CD194<br>CD197<br>CD38<br>CD58<br>CD69<br>IgM	Tax<br>CD45<br>CD183<br>CD95<br>CD9<br>CD68<br>CD11c<br>CD14<br>CD127<br>CD16<br>CD194<br>CD197<br>CD21<br>CD39<br>CD58<br>IgM	CD11c<br>CD194<br>CD3<br>CD8<br>CD38<br>CD39<br>HLA_DR<br>CD5<br>IgM	CD11c<br>CD14<br>CD194<br>CD30<br>CD38<br>CD39<br>HLA_DR<br>CD58<br>CD69<br>CD8a	CD11c<br>CD16<br>CD194<br>CD197<br>CD21<br>CD30<br>CD39<br>CD58<br>IgM
**Summary**	nMo	nMo	unknown	iMo	$CD8^{+}$ T cell	cDC	nMo

Abbreviation
iMo: intermediate monocyte
cDC: dendritic cell
nMo: non-classical monocyte
