# Supplementary material for: HTLV-1 infection of donor-derived T cells might promote acute graft-versus-host disease following liver transplantation
Source: Nat Commun. 2022 Nov 30;13:7368. doi: 10.1038/s41467-022-35111-w (PMC9712688; doi:10.1038/s41467-022-35111-w)
Supplement: Supplementary file 3 — Reporting Summary [file 41467_2022_35111_MOESM3_ESM.pdf]

nature portfolio

Corresponding author(s): Jianjun Zhang, Xianting Ding, and Qiang Xia

Last updated by author(s): 2022/11/10

Reporting Summary

Nature Portfolio wishes to improve the reproducibility of the work that we publish. This form provides structure for consistency and transparency in reporting. For further information on Nature Portfolio policies, see our Editorial Policies and the Editorial Policy Checklist.

Statistics

For all statistical analyses, confirm that the following items are present in the figure legend, table legend, main text, or Methods section.

n/a	Confirmed	
☐	☒	The exact sample size (*n*) for each experimental group/condition, given as a discrete number and unit of measurement
☐	☒	A statement on whether measurements were taken from distinct samples or whether the same sample was measured repeatedly
☐	☒	The statistical test(s) used AND whether they are one- or two-sided<br>*Only common tests should be described solely by name; describe more complex techniques in the Methods section.*
☐	☒	A description of all covariates tested
☐	☒	A description of any assumptions or corrections, such as tests of normality and adjustment for multiple comparisons
☐	☒	A full description of the statistical parameters including central tendency (e.g. means) or other basic estimates (e.g. regression coefficient) AND variation (e.g. standard deviation) or associated estimates of uncertainty (e.g. confidence intervals)
☐	☒	For null hypothesis testing, the test statistic (e.g. *F*, *t*, *r*) with confidence intervals, effect sizes, degrees of freedom and *P* value noted<br>*Give P values as exact values whenever suitable.*
☒	☐	For Bayesian analysis, information on the choice of priors and Markov chain Monte Carlo settings
☒	☐	For hierarchical and complex designs, identification of the appropriate level for tests and full reporting of outcomes
☒	☐	Estimates of effect sizes (e.g. Cohen's *d*, Pearson's *r*), indicating how they were calculated

Our web collection on statistics for biologists contains articles on many of the points above.

Software and code

Policy information about availability of computer code

Data collection	CyTOF v6.7 (analysis software of the Helios mass cytometry and the Hyperion Laser Scanning Module), 7500 software v2.3 (analysis software of ABI 7500 real-time PCR system), Gen 5 v2.09 (analysis software of the BioTek microtiter plate reader)
Data analysis	MCD Viewer v1.0, R 3.5.3, R studio v1.1.463, Cytobank v7.3.0, GraphPad Prism8

For manuscripts utilizing custom algorithms or software that are central to the research but not yet described in published literature, software must be made available to editors and reviewers. We strongly encourage code deposition in a community repository (e.g. GitHub). See the Nature Portfolio guidelines for submitting code & software for further information.

Data

Policy information about availability of data

All manuscripts must include a data availability statement. This statement should provide the following information, where applicable:

- Accession codes, unique identifiers, or web links for publicly available datasets
- A description of any restrictions on data availability
- For clinical datasets or third party data, please ensure that the statement adheres to our policy

The datasets of the TargetSeq and CyTOF analyses and the raw image files of IMC analysis of the study are available in the supplementary information.

Human research participants

Policy information about studies involving human research participants and Sex and Gender in Research.

Reporting on sex and gender	The patient sex was considered into study design, but our findings were not only apply to one sex. The patient sex was determined based on self-reporting and disaggregated sex data was provided in the Table S1 (Patients' information profile). An IMC panel of 19 metal isotope-tagged antibodies was designed to acquire a global overview of the immune cells in the skin lesions and reveal the interactions between HTLV-1 and immune cells. The panel contained 2 structure protein markers, 13 immune cell markers, an HTLV-1 specific marker (Tax protein), and a Y chromosome marker to track the source of cells. IMC was performed on skin lesions from all the 7 patients in the aGVHD group. Among them, 4 female patients (ID139, ID141, ID143, and ID144) received livers from male donors. Thus, eukaryotic translation initiation factor 1A Y-linked (EIF1AY), expressed by the corresponding gene located on Y chromosome, was targeted to track donor-derived cells in samples from female recipients.
Population characteristics	Population characteristics are outlined insupplementary table 1. The donor after circulatory death recipients were divided into two groups, the aGVHD and control groups. The aGVHD group comprised 7 patients diagnosed with aGVHD in different years (2015, 2017, 2018, and 2020). The control group consisted of 17 recipients, including post-transplant rejection patients (N=4), post-transplant infection patients (N=3), post-transplant regular recovery recipients (N=4), and pre-operative patients (N=6). For patients with aGVHD, post-transplant rejection, or post-transplant infection, samples were collected during the disease progression. Specimens were collected before surgery for preoperative control patients. The enrolled patients were adults aged between 18 and 69.
Recruitment	The use of clinical samples was approved by the ethical review board of Renji Hospital, Shanghai Jiao Tong University School of Medicine (clinical trial registration number: KY2019074). All patient samples were obtained with informed consent under the supervision of IRB. The data of their clinicopathological features were anonymized. When GVHD occurs in adult recipients, they can be included in the GVHD group after being confirmed by GVHD diagnosis criteria. All patients diagnosed with aGVHD could be recruited without selection. At the same time, random receptors without GVHD after surgery were recruited to the control group at a ratio of 1:1, and random receptors before surgery were also recruited to the control group at a ratio of 1:1. There were no self-selection bias or other biases.
Ethics oversight	The use of clinical samples was approved by the ethical review board of Renji Hospital, Shanghai Jiao Tong University School of Medicine (clinical trial registration number: KY2019074). All patient samples were obtained with informed consent under the supervision of IRB. The data of their clinicopathological features were anonymized.

Note that full information on the approval of the study protocol must also be provided in the manuscript.

Field-specific reporting

Please select the one below that is the best fit for your research. If you are not sure, read the appropriate sections before making your selection.

☒ Life sciences ☐ Behavioural & social sciences ☐ Ecological, evolutionary & environmental sciences

For a reference copy of the document with all sections, see nature.com/documents/nr-reporting-summary-flat.pdf

Life sciences study design

All studies must disclose on these points even when the disclosure is negative.

Sample size	In discovery cohort, there were 7 patients in the onset group and 17 patients in the control group. The control group was designed as twice the sample size of the onset group. This study is a cross-sectional study with a small sample size. The sample collection method was stratified random sampling and the sample size of each group is generally required to be equal. It is best to increase 10% -20% of the minimum sample requirement in the study. As for the screening cohort, since reported prevalence of HTLV-1 was about 1% and we want to keep the estimation error within 1%, the minimal number of samples is 381 according to normal distribution. The sample size was thus determined as 400 to ensure a reliable estimation of HTLV-1 infection rate in our center.
Data exclusions	No data were artificially excluded from the analysis.
Replication	All attempts at replication were successful. CyTOF and IMC experiments were completed once for all samples. RNA extraction of HTLV-1 was determined to be once a week.
Randomization	Samples were allocated to groups based on disease status (aGVHD and non-aGVHD). All available aGVHD samples in our center during 2017-2020 were included in the aGVHD group. The inclusion of non-aGVHD samples was random and there was no subjective allocation.
Blinding	Blinding is not relevant to our study since there is no clinical intervention involved and no elements that might be influenced by bias from the investigator or observer.

Reporting for specific materials, systems and methods

We require information from authors about some types of materials, experimental systems and methods used in many studies. Here, indicate whether each material, system or method listed is relevant to your study. If you are not sure if a list item applies to your research, read the appropriate section before selecting a response.

Materials & experimental systems

n/a	Involved in the study
☐	☒ Antibodies
☒	☐ Eukaryotic cell lines
☒	☐ Palaeontology and archaeology
☒	☐ Animals and other organisms
☒	☐ Clinical data
☒	☐ Dual use research of concern

Methods

n/a	Involved in the study
☒	☐ ChIP-seq
☒	☐ Flow cytometry
☒	☐ MRI-based neuroimaging

Antibodies

Antibodies used

Anti-CD16 antibody (Abcam, ab215977), Anti-CD11c antibody (Abcam, ab216655), Anti-IgG antibody (Abcam, ab218891), Anti-Collagen antibody (Abcam, ab34710), Anti-CD3 antibody (Abcam, ab5690), Anti-CD14 antibody (Abcam, ab182032), Anti-CD30 antibody (Invitrogen, MA5-12632), Anti-CD68 antibody (Invitrogen, 14-0688-82), Anti-CD21 antibody (Invitrogen, MA5-11417), Anti-α-SMA antibody (Abcam, ab5694), Anti-HLA-DR antibody (Invitrogen, MA1-70112), Anti-CD8a antibody (Abcam, ab213020), Anti-CD4 antibody (Abcam, ab181724), Anti-C4d antibody (Invitrogen, MA5-18045), Anti-EIF1AY antibody (Abcam, ab155546), Anti-TAX antibody (Abcam, ab26997), Anti-CD38 antibody (Abcam, ab176886), Anti-CD123 antibody (Invitrogen, MAB301-100), Anti-CD19 antibody (Invitrogen, 14-0194-82), Anti-CD45 antibody (Fluidigm, 3089003B), Anti-CD196 antibody (Fluidigm, 3141014A), Anti-CD19 antibody (Fluidigm, 3142001B), Anti-CD5 antibody (Fluidigm, 3143007B), Anti-CD195 antibody (Fluidigm, 3144007A), Anti-CD4 antibody (Fluidigm, 3145001B), Anti-CD8 antibody (Fluidigm, 3146001B), Anti-CD25 antibody (Fluidigm, 3149010B), Anti-CD14 antibody (Fluidigm, 3151009B), Anti-CD3 antibody (Biolegend, 300402), Anti-CD194 antibody (Fluidigm, 3158006A), Anti-CD197 antibody (Fluidigm, 3159003A), Anti-CD39 antibody (Fluidigm, 3160004B), Anti-CD80 antibody (Fluidigm, 3161023B), Anti-CD69 antibody (Fluidigm, 3162001B), Anti-CD183 antibody (Fluidigm, 3163004B), Anti-CD95 antibody (Fluidigm, 3164008B), Anti-CD38 antibody (Fluidigm, 3167001B), Anti-CD127 antibody (Fluidigm, 3168017B), Anti-CD9 antibody (Fluidigm, 3171009B), Anti-IgM antibody (Fluidigm, 3172004B), Anti-HLA-DR antibody (Fluidigm, 3174001B), Anti-CD58 antibody (Fluidigm, 3176017B), Anti-CD16 antibody (Fluidigm, 3209002B). Antibody information with dilution is supplied in Supplementary Tables 4 and 5.

Validation

Anti-CD16 antibody (Abcam, ab215977, IHC-P, Human/Rat), Anti-IgG antibody (Abcam, ab218891, IHC-P, Human), Anti-Collagen antibody (Abcam, ab34710, IHC-P, Human/Rat/Mouse), Anti-CD3 antibody (Abcam, ab5690, IHC-P, Human/Rat), Anti-CD14 antibody (Abcam, ab182032, IHC-P, Human/Rat/Mouse), Anti-α-SMA antibody (Abcam, ab5694, IHC-P, Human/Rat/Mouse), Anti-HLA-DR antibody (Invitrogen, MA1-70112, IHC-P, Human), Anti-CD8a antibody (Abcam, ab213020, IHC-P, Human), Anti-CD4 antibody (Abcam, ab181724, IHC-P, Human), Anti-C4d antibody (Invitrogen, MA5-18045, IHC-P, IHC-Fr, Human), Anti-EIF1AY antibody (Abcam, ab155546, IHC-P, Human/Rat), Anti-CD38 antibody (Abcam, ab176886, IHC-P, Human), and Anti-CD19 antibody (Invitrogen, 14-0194-82, IHC-P, Human/Rat/Mouse), Anti-CD30 antibody (Invitrogen, MA5-12632, IHC-P, Flow Cyt, Human), Anti-CD68 antibody (Invitrogen, 14-0688-82, IHC-P, Flow Cyt, Human/Mouse), Anti-CD21 antibody (Invitrogen, MA5-11417, IHC-P, Flow Cyt, Human), and Anti-CD123 antibody (Invitrogen, MAB301-100, IHC-P, Flow Cyt, Human), Anti-TAX antibody (Abcam, ab26997, IHC-P, IHC-Fr, HTLV-1) and Anti-CD11c antibody (Abcam, ab216655, IHC-P, IHC-Fr, Human), Anti-CD45 antibody (Fluidigm, 3089003B, CyTOF, Human), Anti-CD196 antibody (Fluidigm, 3141014A, CyTOF, Human), Anti-CD19 antibody (Fluidigm, 3142001B, CyTOF, Human), Anti-CD5 antibody (Fluidigm, 3143007B, CyTOF, Human), Anti-CD195 antibody (Fluidigm, 3144007A, CyTOF, Human), Anti-CD4 antibody (Fluidigm, 3145001B, CyTOF, Human), Anti-CD8 antibody (Fluidigm, 3146001B, CyTOF, Human), Anti-CD25 antibody (Fluidigm, 3149010B, CyTOF, Human), Anti-CD14 antibody (Fluidigm, 3151009B, CyTOF, Human), Anti-CD3 antibody (Biolegend, 300402, CyTOF, Human), Anti-CD194 antibody (Fluidigm, 3158006A, CyTOF, Human), Anti-CD197 antibody (Fluidigm, 3159003A, CyTOF, Human), Anti-CD39 antibody (Fluidigm, 3160004B), Anti-CD80 antibody (Fluidigm, 361023B, CyTOF, Human), Anti-CD69 antibody (Fluidigm, 3162001B, CyTOF, Human), Anti-CD183 antibody (Fluidigm, 3163004B, CyTOF, Human), Anti-CD95 antibody (Fluidigm, 3164008B, CyTOF, Human), Anti-CD38 antibody (Fluidigm, 3167001B, CyTOF, Human), Anti-CD127 antibody (Fluidigm, 3168017B, CyTOF, Human), Anti-CD9 antibody (Fluidigm, 3171009B, CyTOF, Human), Anti-IgM antibody (Fluidigm, 3172004B, CyTOF, Human), Anti-HLA-DR antibody (Fluidigm, 3174001B, CyTOF, Human), Anti-CD58 antibody (Fluidigm, 3176017B, CyTOF, Human), and Anti-CD16 antibody (Fluidigm, 3209002B, CyTOF, Human). Antibody information with reactivity species is supplied in Supplementary Tables 4. Primaries antibodies of IMC panel were validated on FFPE sections by using REAL EnVision kit (DAKO) and the manufacturer's websites stated that antibodies were validated on FFPE by immunohistochemistry. Primaries antibodies of CyTOF panel were validated on PBMCs by CyTOF and the manufacturer's websites stated antibodies for CyTOF panel were validated by immunofluorescence, flow cytometry, or CyTOF.
